# With or Without Nasal Continuous Positive Airway Pressure During Delayed Cord Clamping in Premature Infants <32 Weeks: A Randomized Controlled Trial Using an Intention-To-Treat Analysis

**DOI:** 10.3389/fped.2022.843372

**Published:** 2022-03-31

**Authors:** Rui Deng, Yan Wu, Guiyuan Xiao, Xiaoyun Zhong, Hua Gong, Wen Chen, Ligang Zhou, Biao Shen, Qi Wang

**Affiliations:** ^1^Neonatal Department, Chongqing Health Center for Women and Children, Chongqing, China; ^2^Chongqing Health Center for Women and Children, Chongqing, China

**Keywords:** delayed cord clamping, nCPAP (nasal CPAP), preterm, resuscitation, ventilation

## Abstract

**Objective:**

To assess whether providing nasal continuous positive airway pressure (nCPAP) during delayed cord clamping is beneficial for preterm infants <32 weeks.

**Study Design:**

A randomized controlled trial was performed from March 2020 to May 2021. Premature infants (<32 weeks of gestational age; *n* = 160) were allocated to receive at least 60 s of delayed cord clamping with nCPAP (DCC+nCPAP; *n* = 80) or without nCPAP (DCC only; *n* = 80). For both groups, after the umbilical cord was clamped, the infants were carried immediately to the resuscitation room to continue receiving standard transition. The primary outcome was the mechanical ventilation (MV) rate within 24 h of life. The measurements related to early respiratory support effect before cord clamping including positive end-expiratory pressure (PEEP) and FiO_2_ during transition/leaving the delivery room, intubation rate during transition, pulmonary surfactant (PS) administration ≥2 times after birth, extubation failure, and incidence of bronchopulmonary dysplasia (BPD) were collected as the secondary outcomes. Furthermore, other neonatal short-term outcomes and safety assessment were also included.

**Results:**

The measurements were calculated using intention-to-treat analysis. The median time for cord clamping were 60 s with interquartile range (IQR) (60.00–60.00 vs. 60.00–70.00) in both groups. There were no difference in the primary outcome of MV rate within 24 h of life (*p* = 0.184). The arterial blood gas pH at 1 h after birth in the DCC+nCPAP group was 7.28 ± 0.08 vs. 7.25 ± 0.07 in the control group (mean difference = 0.01, 95% CI: −0.01–0.05, *p* = 0.052), which approached statistical significance. There was no significant statistical difference in the other short-term neonatal outcomes and the safety indicators between the two groups.

**Conclusions:**

Our study showed that delayed cord clamping with nCPAP was feasible and safe in preterm infants with gestational age <32 weeks. Although there was a trend toward a higher arterial blood gas pH at 1 h after birth in the DCC+nCPAP group, DCC+nCPAP neither resulted in a corresponding measurable clinical improvement nor did it reduce subsequent neonatal morbidity. A larger multi-center study including more infants with gestational age <28 weeks is needed to evaluate the full effects of DCC in combination with nCPAP in preterm infants.

## Introduction

Although there has been many recent advances in perinatal medicine, it was still a challenge to manage the effective care of extremely preterm infants in the delivery room.

In recent years, there has been increasing evidence to support that, when compared with immediate cord clamping (ICC), delayed cord clamping (DCC) increases placental transfusion and the infants' blood volume, reduces later anemia (1, 2), and decreases the incidence of intraventricular hemorrhage (IVH) (3), late-onset sepsis, and necrotizing enterocolitis (NEC) in premature infants (4, 5). Thus, the International Liaison Committee on Resuscitation (ILCOR) argued that the umbilical cord of the infants who do not need resuscitation should not be clamped until at least 1 min after birth (6).

However, in previous studies, the majority of preterm infants requiring respiratory stabilization at birth was excluded because some neonatologists were worried that if respiratory support could not be offered to these infants as soon as possible, their lung aeration and the establishment of the spontaneous breathing would be affected. Therefore, those infants with breathing difficulties were separated from the placenta earlier and with subsequent intervention.

In fact, most extremely preterm infants might inevitably need early respiratory support in order to help them complete their physiological transition and lung aeration (7). Theoretically, continuous positive end-respiratory pressure (PEEP), which could be provided by nasal continuous positive airway pressure (nCPAP), could contributed to the early physiological transition by promoting pulmonary fluid absorption, facilitating alveolar recruitment, and establishing functional residual capacity (FRC) in premature infants. However, the question as to whether providing nCPAP before cord clamping could improve the respiration of preterm infants still remained unanswered.

Our hypothesis was that providing nCPAP during DCC could contribute to the early physiological transition by establishing an adequate FRC, thus improving lung compliance and the pulmonary gas exchange. This could decrease the work of breathing and reduce the need for a higher PEEP and the fractional of inspiration O_2_ (FiO_2_). In turn, this would reduce the rate of mechanical ventilation (MV) for premature infants during the early postnatal period. We, therefore, conducted a randomized controlled trial (RCT) to compare the outcomes of DCC with nCPAP (DCC+nCPAP) and DCC without nCPAP (DCC-only). The MV rate within the first 24 h of life was measured as the primary outcome. Other measurements served as the secondary outcomes.

## Methods

### Participants

An RCT involving 145 mothers/160 infants was undertaken from March 2020 to May 2021. Women who expected to have a live birth before 32 weeks of gestation were eligible, regardless of the infants' birth mode and fetal presentation. The exclusion criteria were as follows: (1) parents who refused to sign the informed consent; (2) maternal factors, such as a mother who needed general anesthesia and those who had placental abruption, placenta previa, monochorionic twins, and triplets; (3) fetal factors, such as when any major congenital abnormalities were diagnosed prenatally in any infant, or the presence of either twin–twin transfusion syndrome or hydrops; and (4) any other situations that might endanger the safety of the mothers and infants involved in the study.

### Interventions

Immediate nCPAP with DCC was compared with DCC only. The delay time of umbilical cord ligation in both groups was at least 60 s. DCC for at least 60 s was based on the balance between the time acceptable to the obstetricians and/or neonatologists and the recommendation of waiting for at least 30–60 s made by the European Consensus Guidelines, the American College of Obstetricians and Gynecologists, and most of the study protocols detailed in several systematic reviews (8–11). The temperature of either the delivery room or operating room was pre-adjusted to 28–30°C. Preheated towels were used to dry the newborn infant as quickly as possible, and these were replaced with new ones to in order prevent heat loss during the intervention. The infants were placed onto a firm surface with easy access to resuscitation equipment and were kept at the placental level until the umbilical cord was clamped. When the infant was randomized to the DCC+nCPAP group, nCPAP would be given with the cord intact immediately after delivery. In order to avoid any contamination, the neonatologist would have scrubbed up and put on a gown before the infant was born. Then, a pair of suitably sized bi-nasal prongs would be connected to a ventilator (HAMILTON- C1, Hamilton Medical AG, Switzerland, *via* a Crusch 8 7402 Bonaduz, Schweiz) with the tube being placed in a transparent plastic sleeve in advance. The binasal prongs, tubes, and sleeves were all pre-sterilized. The infant would be stimulated by gently rubbing of the back with warm sterile towels in both groups. All infants included in the study were connected to a pulse oximeter on both the right wrist and right foot, and an electrocardiogram was performed immediately after birth. Cord milking was not performed in either group. A clock was used for timing.

Under some special circumstances when the umbilical cord was too short for DCC+nCPAP, or the infants needed intubation or maternal emergency, the umbilical cord would be clamped within 60 s. For both groups, after the umbilical cord was clamped, the infants were carried immediately to an independent resuscitation room in order to continue standard resuscitation. The neonatal team (blinded to the intervention) resuscitated/transitioned the infants in accordance with the 2015 US Neonatal Resuscitation Guidelines (12). Standard equipment such as plastic bags, towels, and hats were used routinely. In addition, a Giraffe incubator (Giraffe Incubator Carestation SC1, Ohmeda Medical, United States) and Giraffe shuttle (Giraffe shuttle, Ohmeda Medical, United States) were used for resuscitation/transition and intra-hospital transfers.

In the delivery room, according to our unit's protocol, the initial CPAP was 6 cm H_2_O; the initial FiO_2_ was 0.30 for infants <28 weeks' gestation and 0.21–0.30 for those at 28–31 weeks during transition. For persistently apneic or bradycardic (<100 bpm) infants, positive pressure ventilation (PPV) was used with a starting peak inspiratory pressure (PIP) of 20 cm H_2_O and PEEP of 6 cm H_2_O, respectively. The less invasive surfactant administration (LISA) method (13) was used for pulmonary surfactant (PS) administration in the delivery room for infants <28 weeks gestation in case that intubation was not required. All the doctors who used this procedure were highly trained and experienced. If PPV was ineffective or a long time was needed for PPV or there was a need of chest compression, the intubate–surfactant–extubate (INSURE) method was considered. The surfactant was administrated through the endotracheal tube; if the FiO_2_ dropped to 30% in a short time without dyspnea, then the extubation and non-invasive respiratory support was continued. If not, MV was be implemented.

In the neonatal intensive care unit (NICU), if the CPAP pressure ≥6 cm H_2_O with FiO_2_ >0.30 and the infant's dyspnea gradually worsened, the LISA method would be considered. If the infants are with severe respiratory distress syndrome (RDS), dyspnea rapid progressed and/or persisted, FiO_2_ ≥40%, arterial oxygen pressure (PaO_2_) <50–60 mmHg, or oxygen saturation (SpO_2_) <90%, the INSURE method would be considered. The initial Poractant alfa (Curosurf, Chiesi Farmaceutici S.p.A., Via Palermo 26/A-43122 Parma, Italy) dose was 200 mg/kg. If the RDS was severe and the improvement was not apparent after the first PS treatment or the infants' condition worsened after a remission, PS would be given 6–12 h later. The protocol for MV in preterm infants with gestational age <32 weeks was as follows: (1) in infants <32 weeks' gestation with severe apnea (defined as recurrent apnea with >3 episodes/h associated with a heart rate <100/min or a single episode of apnea that required PPV, or an associated with SpO_2_ <85% and FiO_2_ >0.6); (2) in infants with RDS, dyspnea rapidly progressed and/or persisted after non-invasive ventilation and/or PS treatment, and FiO_2_ ≥40%, PaO_2_ <50–60 mmHg, or SpO_2_ <90% (except for cyanotic heart disease) or PCO_2_ >60–65 mmHg, pH <7.20; (3) in cases where general anesthesia was required; and (4) there was some instability in the hemodynamics of the infants.

### Outcome Measures

The primary outcome was MV rate within 24 h of life in each group, which related to the early respiratory support effect before cord clamping.

#### Secondary Outcomes

1) Outcomes concerning the transition of the infant: (1.1) intubation, (1.2) maximum PEEP, (1.3) PEEP when leaving the delivery room, (1.4) maximum FiO_2_, (1.5) FiO_2_ when leaving the delivery room, (1.6) requirement for PPV, (1.7) SpO_2_ <80% 5 min after birth, (1.8) heart rate (HR) <100 bpm 5 min after birth, (1.9) rectal temperature at admission, (1.10) rectal temperature <36.0°C, (1.11) SpO_2_ at 5 and 10 min, (1.12) HR at 5 and 10 min, (1.13) Apgar scores at 1, 5, and 10 min, and (1.14) arterial blood gas (ABG) hydrogen ion concentration (pH) and lactate (LAC) at 1 h after birth.
2) Short-term neonatal outcomes: (2.1) PS ≥2 times after birth, (2.2) extubation failure, (2.3) duration need for oxygen in the NICU, (2.4) frequency of phototherapy, (2.5) peak hematocrit (Hct) in the first 12–24 h, (2.6) polycythemia (Hct >65%), (2.7) BPD, (2.8) late-onset sepsis, (2.8) IVH ≥ grade 3, (2.10) periventricular leukomalacia (PVL), (2.11) retinopathy of prematurity (ROP, ≥phase 2), (2.12) NEC, ≥ phase 2, and (2.14) death during hospitalization.
3) Short-term maternal outcomes: (3.1) postpartum hemorrhage ≥1, 000 ml and (3.2) postpartum infection rate.

Safety assessment: (1) SpO_2_ (<80%) at 5 min, (2) HR (<100 bpm) at 5 min, (3) rectal temperature at admission (<36.0°C), (4) postpartum hemorrhage ≥1, 000 ml, and (5) postpartum infection rate.

Definition of the important diagnoses and concepts used: BPD was defined as premature infants with a gestational age <32 at 36 weeks postmenstrual age (PMA), requiring different degrees of FiO_2_ for 3 consecutive days to maintain arterial oxygen saturation in the range of 90–95%, and having persistent parenchymal lung disease confirmed by radiographic evidence (14). Late-onset sepsis was defined as a positive culture (including bacterium or fungus) in blood or cerebrospinal fluid in infants older than 3 days with clinical signs of infection (15). IVH classification was according to Papile et al. (16). PVL was defined by serial head ultrasound, according to the description by Volpe (17). ROP classification was according to Alice (18) and for NEC; Bell staging was used (19).The diagnosis of RDS was based on clinical manifestations and chest X-ray (20).

### Recruitment and Randomization

The trial has been approved by the Ethics Committee of the Chongqing Health Center for Women and Children.

The study was registered at chictr.org.cn (ChiCTR2000029910). Women at risk of preterm birth at <32 weeks gestation were invited to participate in the study, and each participant gave a written consent. Eligibility and consent were verified before randomization, which was conducted before either the vaginal delivery or cesarean section. Before delivery, participants were randomized by opaque sequentially numbered sealed envelopes, which were computer generated. The members of the research team opened the envelope after receiving the notification that the subject was about to give birth and reviewed the protocol with the obstetrician. The time was subsequently recorded, and the time from the delivery to the umbilical cord clamping of the two groups was recorded, too. In the case of twins, it was difficult to perform DCC with nCPAP for both infants. After consent, the first infant would be randomly assigned to either the DCC+nCPAP group or DCC-only group, and the second baby would be automatically assigned to the other group.

### Blinding

The blind method could not be implemented during DCC (with or without nCPAP). However, the treatment allocation plan of the follow-up resuscitation personnel in the special resuscitation room and the medical staff taking care of patients after admission were blinded to the treatment used. Because the end point needed to be recorded by an evaluator who was not involved in patient care, this person was blinded to the treatment regimen used and could review the patient files that mask the type of treatment. In addition, the investigator who performed the final statistical analysis did not know the treatment allocation.

### Sample Size

According to our preliminary study, it was estimated that the rate of MV within 24 h of life in the control group was about 25.8%, and that in the intervention group, it was 8%.Taking α = 0.05 and the test power (1–β) = 0.8, according to the sample size calculation formula, the minimum sample size of the comparison rate between the two groups was about 138 cases. This translated to a minimum of 69 cases in each group, and the loss to follow-up rate of each group was estimated to be 15%. Therefore, this study had 80 infants each in the intervention and control groups with a total sample size of 160 cases.

### Statistical Analysis

In order to reduce the risk of bias, intention-to-treat analysis was used to assess the results. The data from all the infants were analyzed based on the group to which they were randomly assigned, regardless of the practical timing of cord clamping. Infants were randomly assigned to DCC+nCPAP group and DCC-only group. The data were tested for normality using the Shapiro–Wilk test. Normally distributed quantitative data were represented with $\bar{\chi }\;\pm \;s$, and the Student's *t*-test was adopted to perform the comparison between groups. Non-normally distributed quantitative data were expressed as M (P25, P75), using the Wilcoxon Mann–Whitney rank sum test for the comparison among the groups. *n* (%) was used to qualitative data when the *X*^2^-test was used for the comparison. The difference in risk effects was presented by mean difference, median difference, and the relative risk (RR), of which the calculation method used were the two-sample *t*-test, quantile regression, and logistic regression, respectively. A *p*-value of < 0.05 was considered statistically significant. Statistical analyses were performed with the use of SAS and SPSS software packages, versions 9.21 and 25.0, respectively.

## Results

**Figure 1:**
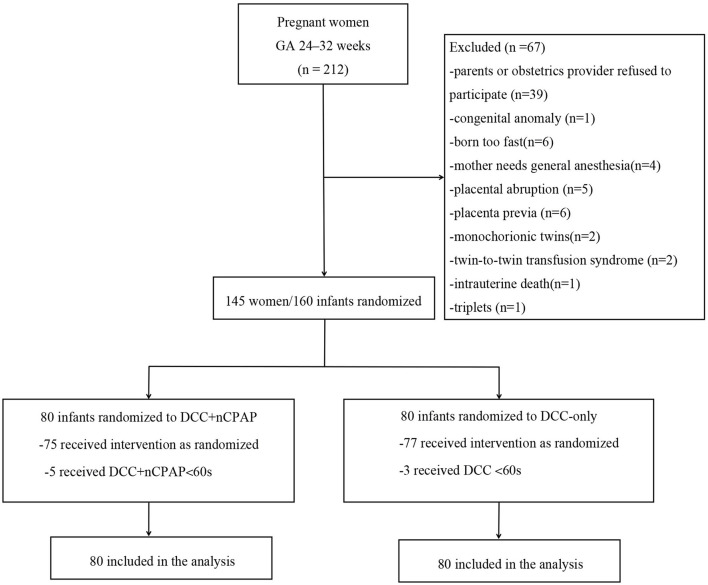
Flow diagram of patients in the study.

Recruitment started in March 2020 and ended in May 2021. During the study period, a total of 212 women were admitted and went into labor at GA of 24–32 weeks. Among them, 67 women were excluded, and 145 pregnant women/160 infants were enrolled. Eighty infants were allocated to the DCC+nCPAP group while 80 infants to the DCC-only group. Five infants in the DCC+nCPAP group did not receive DCC for at least 60 s (three due to umbilical cord being too short and two needed intubation), and three infants in the DCC-only group did not receive DCC for at least 60 s (one needed intubation and two due to maternal emergency). Among the 160 infants, 2 died in the DCC+nCPAP group died (1 with NEC stage 3 and intestinal perforation and 1 with severe acidosis/pulmonary hemorrhage), and 3 infants died in the DCC-only group (1 with disseminated intravascular coagulation and shock, 1 with pulmonary hemorrhage, and 1 with severe acidosis) (Figure 1).

The mean gestational age in the DCC+nCPAP group and DCC-only group were 29 + 6 weeks and 30 + 1 weeks, respectively. There were 42 (52.5%) males in the DCC+nCPAP group and 49 (61.25%) in the control group. Sixty-four (80%) cases underwent cesarean section in the intervention group and 60 (75.00%) in the control group. The mean birth weights were 1, 325.71 ± 332.08 g and 1, 411.13 ± 332.87 g in the DCC+nCPAP and control group, respectively. All the other baseline characteristics of the mothers and infants noted in these two groups were not significant different (Table 1).

**Table 1:** Baseline characteristics.

	**DCC+nCPAP** **(***n*** = 80)**	**DCC-only** **(***n*** = 80)**	***t/X*** **^2^/** ***Z***	***p*** **-value**
Gestational age (weeks) (IQR)	29.84 (28.00, 31.14)	30.14 (28.84, 31.28)	1.238	0.216
<28 weeks *n* (%)	18 (22.50)	11 (13.75)	2.064	0.151
28–31 + 6 weeks *n* (%)	62 (77.50)	69 (86.25)		
Birth weight (gram) (*x* ±*s*)	1, 325.71 ± 332.08	1, 411.13 ± 332.87	1.625	0.106
Male (*n*) (%)	42 (52.50)	49 (61.25)	1.249	0.264
Cesarean section (*n*) (%)	64 (80.00)	60 (75.00)	0.573	0.449
Standard of dexamethasone usage (*n*) (%)	55 (68.75)	57 (71.25)	0.119	0.730
Standard of magnesium sulfate usage (*n*) (%)	59 (73.75)	54 (67.50)	0.753	0.385
Antibiotics (*n*) (%)	36 (45.00)	41 (51.25)	0.626	0.527
Pregnancy-induced hypertension (*n*) (%)	9 (11.25)	7 (8.75)	0.278	0.598
ICP (*n*) (%)	3 (3.75)	1 (1.25)	1.026	0.311
PROM (*n*) (%)	35 (43.75)	34 (42.50)	0.025	0.873
Chorioamnionitis (*n*) (%)	10 (12.50)	11 (13.75)	0.055	0.815
GBS (+) (*n*) (%)	44 (55.00)	51 (63.75)	1.701	0.427
GDM (*n*) (%)	27 (33.75)	21 (26.25)	1.071	0.301
Subclinical hypothyroidism during Pregnancy (*n*) (%)	6 (7.50)	5 (6.25)	0.098	0.755
DCC (s) (IQR)	60.00 (60.00, 60.00)	60.00 (60.00, 70.00)	1.436	0.151

*IQR, interquartile range; ICP, intrahepatic cholestasis of pregnancy; PROM, premature rupture of membranes; GBS, group B streptococcus; GDM, gestational diabetes mellitus; DCC, delayed cord clamping*.

The median time for cord clamping was 60 s both in the DCC+nCPAP group [interquartile range (IQR), 60–60] and the control group (IQR, 60–70), *p* = 0.151 (Table 1).

### Primary Outcome

Nine infants (11.25%) in the DCC+nCPAP group and 15 (18.75%) infants in the DCC only group underwent MV within 24 h of life. There was no difference in the primary outcome of MV within this time period (RR = 0.549, 95% CI: 0.225–1.341, *p* = 0.184) (Table 3). For infants <28 weeks, there was no significant difference in the MV rate within 24 h of life, 4/18 (22.22%) vs. 5/11 (45.45%) (RR = 0.343, 95% CI: 0.067–1.743, *p* = 0.189).

### Secondary Outcomes

The ABG pH at 1 h after birth in the DCC+nCPAP group was 7.28 ± 0.08, while it was 7.25 ± 0.07 in the control group with the *p*-value of 0.052 (mean difference = 0.01, 95% CI: −0.01–0.05). This approached statistical significance (Table 2).

**Table 2:** Outcomes concerning the transition of the infants.

**Outcomes concerning the transition of the infants**	**DCC+nCPAP** **(***n*** = 80)**	**DCC-only** **(***n*** = 80)**	**Difference** **(95% CI)^a^**	***t//X*** **^2^/** ***Z***	***p*** **-value**
Intubation in transition (*n*) (%)	5 (6.25)	5 (6.25)	1.000 (0.278–3.598)^b^	0.000	1.000
Max PEEP during transition (cmH_2_O) (IQR)	9.00 (8.00, 10.00)	8.0 (7.00, 10.00)	0.00 (0.00–1.00)^c^	0.603	0.547
PEEP when leaving the delivery room (cmH_2_O) (IQR)	6.00 (6.00, 7.00)	6.00 (6.00, 7.00)	0.00 (0.00–0.00)^c^	0.167	0.867
Max FiO_2_ during transition (%) (IQR)	40.00 (30.00, 60.00)	40.00 (30.00, 55.00)	0.00 (−5.00–5.00)^c^	0.235	0.814
FiO_2_ leaving the delivery room (%) (IQR)	30.00 (25.00, 35.00)	30.00 (25.00, 35.00)	0.00 (0.00–0.00)^c^	0.598	0.550
PPV required* (*n*) (%)	10 (12.50)	9 (11.25)	1.127 (0.432–2.941)^b^	0.060	0.807
SpO_2_ <80% at 5 min (*n*) (%)	12 (15.00)	13 (16.25)	0.910 (0.387–2.137)^b^	0.047	0.828
HR <100 bpm at 5 min (*n*) (%)	1 (1.25)	3 (3.75)	0.352 (0.036–3.461)^b^	0.182	0.350
Admission temperature (°C) (*x* ±*s*)	36.96 ± 0.35	36.95 ± 0.33	0.13 (−0.09–0.12)^d^	0.232	0.817
Admission temperature (<36.0°C) *n* (%)	0	0	—	—	—
SpO_2_ at 5 min (%) (IOR)	82.00 (80.00, 85.00)	82.00 (80.00, 85.75)	0.00 (−2.00–1.00)^c^	0.597	0.550
SpO_2_ at 10 min (%) (IQR)	92.00 (89.50, 93.00)	92.00 (90.00, 94.00)	0.00 (−1.00–1.00)^c^	0.536	0.592
HR at 5 min (bpm) (*x* ±*s*)	142.00 ± 19.19	144.49 ± 19.73	−3.15 (−9.45–3.15)^d^	0.988	0.325
HR at 10 min (bpm) (*x* ±*s*)	154.94 ± 14.93	154.25 ± 20.05	0.40 (−5.39–6.19)^d^	0.135	0.892
Apgar score at 1 min (IQR)	9.00 (8.00, 10.00)	9.00 (8.00, 10.00)	0.00 (−1.00–0.00)^c^	1.249	0.212
Apgar score at 5 min (IQR)	9.00 (9.00, 10.00)	10.00 (9.00, 10.00)	0.00 (0.00–0.00)^c^	1.360	0.174
Apgar score at 10 min (IQR)	10.00 (9.00, 10.00)	10.00 (9.00, 10.00)	0.00 (0.00–0.00)^c^	0.626	0.531
ABG pH 1 h after birth (*x* ±*s*)	7.28 ± 0.08	7.25 ± 0.07	0.01 (−0.01–0.05)^d^	1.969	0.052
ABG LAC at 1 h after birth (mmol/L) (IQR)	1.30 (1.00, 2.05)	1.3 (0.90, 2.00)	0.00 (−0.10–0.30)^c^	0.667	0.505

*PEEP, positive end-expiratory pressure; FiO_2_, fraction of inspiration O_2_; IQR, interquartile range; PPV, positive pressure ventilation; SpO_2_, oxygen saturation; HR, heart rate; BPM, beat per minute; ABG, arterial blood gas; pH, hydrogen ion concentration*.

* *Non-intubation*.

a *“Difference” means the difference of risk effects and was presented by mean difference, median difference, and the RR (relative risk), of which calculation method were two-sample t-test, quantile regression, and logistic regression, respectively*.

b *RR (relative risk)*.

c *Median difference*.

d *Mean difference*.

The duration of MV (hours) during the hospitalization in both groups was not significantly different (52 vs. 48, median difference = −8.75, 95% CI: −61.50–34.00, *p* = 0.681). There was no significant difference in the outcome of intubation rate during transition in both groups, 5 (6.25%) vs. 5 (6.25%) (RR = 1.95% CI: 0.278–3.598, *p* = 1.00) (Table 2). There was no difference between the two groups in the maximum PEEP during transition (9 vs. 8, median difference = 0.95% CI: 0.00–1.00, *p* = 0.547). There were no differences between the two groups in PEEP when leaving the delivery room (6 vs. 6, median difference = 0.95% CI: 0.00–0.00, *p* = 0.867). There was no difference between the two groups in maximum FiO_2_ during transition (40 vs. 40, median difference = 0.95% CI: −5.00–5.00, *p* = 0.814). There were no difference between the two groups in FiO_2_ when leaving the delivery room (30 vs. 30, median difference = 0.95% CI: 0.00–0.00, *p* = 0.550) (Table 2). There was no significant difference between the two groups in PS administration ≥2 times after birth, 0 (0.00%) vs. 3 (3.75%) (*p* = 0.245). There was no significant difference in the incidence of extubation failure between the two groups, 0 (0.00%) vs. 2 (2.5%) (*p* = 0.497). There was also no significant difference between the two groups regarding the incidence of BPD (all grade), 2 (2.5%), vs. 8 (10.00%) (RR = 0.231, 95% CI: 0.047–1.123, *p* = 0.102) (Table 3). There were no differences between the two groups in any of the other outcomes.

**Table 3:** Short-term neonatal outcomes.

**Short-term neonatal outcomes**	**DCC + nCPAP** **(***n*** =8 0)**	**DCC-only** **(***n*** = 80)**	**Difference** **(95% CI)^a^**	***t//X*** **^2^/** ***Z***	***p*** **-value**
MV within 24 h of life (*n*) (%)	9 (11.25)	15 (18.75)	0.549 (0.225–1.341)^b^	1.765	0.184
PS ≥2 times (*n*) (%)	0 (0.00)	3 (3.75)	—	— (Fisher test)	0.245
Extubation failure (*n*) (%)	0 (0.00)	2 (2.50)	—	— (Fisher test)	0.497
Duration of oxygen in the NICU (days) (IQR)	22.00 (8.50, 40.50)	18.00 (6.00, 36.00)	2.00 (−4.00–6.00)^c^	0.097	0.922
Frequency of phototherapy (times) (IQR)	4.00 (2.00, 6.00)	4.00 (3.00,6.00)	0.00 (−0.80, 0.30)^c^	0.908	0.364
Peak Hct in the first 12–24 h (%) (*x* ±*s*)	56.91 ± 9.82	58.46 ± 8.88	−1.55 (−4.53–1.43)^d^	1.026	0.307
(Hct > 65% in the first 24 h (*n*) (%)	18 (22.50)	22 (27.50)	0.765 (0.373–1.570)^b^	0.533	0.465
BPD (≥grade 2) (*n*) (%)	0	0	—	—	—
BPD (all grade) (*n*) (%)	2 (2.50)	8 (10.00)	0.231 (0.047–1.123)^b^	2.667	0.102
Late-onset sepsis (*n*) (%)	1 (1.25)	3 (3.75)	0.325 (0.033–3.192)^b^	0.256	0.613
IVH ≥ grade 3 (*n*) (%)	1 (1.25)	1 (1.25)	1.000 (0.061–16.270)^b^	— (Fisher test)	1.000
PVL (*n*) (%)	1 (1.25)	0 (0.00)	—	— (Fisher test)	1.000
ROP ≥ phase 2 (*n*) (%)	9 (11.25)	7 (8.75)	1.322 (0.467–3.741)^b^	0.278	0.598
NEC ≥ phase 2 (*n*) (%)	5 (6.25)	2 (2.50)	2.600 (0.489–13.814)^b^	0.598	0.440
Death (*n*) (*n*) (%)	2 (2.50)	3 (3.75)	0.658 (0.107–4.048)^b^	0.206	0.650

*IQR, interquartile range; Hct, hematocrit; BPD, bronchopulmonary dysplasia; IVH, intraventricular hemorrhage; PVL, periventricular leukomalacia; ROP, retinopathy of prematurity; NEC, necrotizing enterocolitis*.

a *“Difference” means the difference of risk effects and was presented by mean difference, median difference, and the RR (relative risk), of which calculation method were two-sample t-test, quantile regression, and logistic regression, respectively*.

b *RR (relative risk)*.

c *Median difference*.

d *Mean difference*.

### Safety Assessment

There was no statistical significance in any of the safety indicators between the two groups (*p* > 0.05). In the DCC+nCPAP group, the incidence rate of SpO_2_ <80% within 5 min after birth was 15.0%, and there was only one infant (1/80, 1.25%) with a HR <100 bpm at 5 min after birth. In both groups, the rates of postpartum infection and postpartum hemorrhage were 2.5%. The incidence rate of a rectal temperature of <36.0°C in the infants at the time of admission was zero. In addition, the mean admission rectal temperature of infants in the two groups was ≥36.5°C (Tables 2, 3).

## Discussion

Theoretically, PEEP provided by nCPAP could contribute to the early physiological transition by promoting pulmonary fluid absorption, establishing FRC and improving lung compliance and the pulmonary gas exchange in extremely premature infants before cord clamping. In addition, the negative pressure in the pleural cavity generated by effective respiration during DCC could increase the power of blood flowing from the placenta to the infants. This, in turn, may result in increased blood volume, less fluctuation in blood flow, and a decreased incidence of IVH. Although it was recommended by WHO that those with experience should initiate positive pressure ventilation before cutting the umbilical cord (21), there have been only a few studies related to DCC with nCPAP during transition in premature infants reported recently. This single-center analysis evaluated the feasibility and effectiveness of DCC with nCPAP for preterm infants <32 weeks under the existing equipment conditions in one particular unit.

However, in this study, we found no significant difference in the primary outcome of the MV rate within 24 h of life between infants undergoing DCC+nCPAP and DCC only, with a result of 9 (11.25%) vs. 15 (18.75%) (RR = 0.549, 95% CI: 0.225–1.341, *p* = 0.184), respectively (Table 3). In addition, the infants < 28 weeks showed no significant difference in the MV rate, 4/18 (22.22%) vs. 5/11 (45.45%) (RR = 0.343, 95% CI: 0.067–1.743, *p* = 0.189). The duration of MV (hours) during the hospitalization in both groups presented no significant difference (52 vs. 48, median difference = −8.75, 95% CI: −61.50–34.00, *p* = 0.681), which was consistent with a study by Katheria et al. (22). In addition, there were no significant differences in terms of the maximum PEEP and FiO_2_ during transition, the respective values at the time of leaving the delivery room and the intubation rate during transition between the two groups. In the intervention group, no infant needed PS for a second time, and no infant failed to extubate, while in the DCC-only group, the figures were 3/(3.75%) and 2/(2.5%), respectively. However, the differences above were not significant (*p* > 0.05). Therefore, our research did not show that providing nCPAP before umbilical cord clamping would significantly improve the respiratory related measurements.

In practice, the majority of the extremely preterm infants with gestational age of <28 weeks tended to experience respiratory distress and needed more respiratory support immediately after birth. This was probably due to their structurally immature lungs, deficiencies in surfactant levels, weak intercostal muscles, and poor diaphragmatic functions, and difficulty to establish and maintain a functional residual capacity. However, in this study, the mean gestational age in the DCC+nCPAP group and DCC-only group were 29 + 6 week and 30 + 1 week, respectively. The gestational ages of most of the infants were generally more than 28 weeks (81.9%). These infants tended to establish effective breathing easier than the infants with smaller gestational ages. Although due to limitations with respect to readily available equipment, we were not able to accurately record the time points when the infants in both groups started breathing and achieved stable breathing during the DCC period. However, the average time period for first breathing in a similar study (22) was <60 s, which was earlier than the average time for cord clamping in our study. In addition, it was important to point out that in their study, the average gestational age of the preterm infants was about 28 weeks, which was less than that in our study. Hence, we speculated that most of the infants in both groups might have established the same respiratory effect at the average time of cord clamping in our study. Therefore, there was no significant difference between the two groups in terms of the respiration-related outcomes. It would have been a worthwhile exercise to find out the difference between DCC+ nCPAP and DCC-only by including more infants with gestational ages of <28 weeks. However, in the present study, there were only 29 (18.1%) extremely premature infants <28 weeks that accounted for a relatively small proportion of the total. Hence, it was possible that the results obtained might not make a difference. We speculated that more beneficial results could be obtained if more infants with gestational age <28 weeks would be included. In addition, it was presented in the study of Katheria et al. (22) that offering gentle tactile stimulation during DCC may prompt the establishment of spontaneous respiration and supply a similar placental transfusion when compared to CPAP ± PPV. Hence, we speculated that gentle tactile stimulation might play a similar role in promoting effective respiratory establishment as nCPAP during DCC, although it was performed in both groups in this study.

It was worth noting that if the timing of DCC+nCPAP was not long enough to allow sufficient lung aeration, a stable and effective spontaneous breathing might not be fully established in some infants before the cord was clamped. A previous clinical study reported that neonatal mortality was found to be higher if the umbilical cord was ligated before spontaneous breathing started (23). Nevill and Meyer (24) pointed out that DCC could lead to insufficient placental blood transfusion in newborns who were unable to breathe effectively. It was possible that more beneficial results could be obtained if the duration of DCC+ nCPAP was extended appropriately, depending on the individual circumstance. Based on the physiological characteristics, Knol et al. (25) proposed a model in which respiratory support and DCC could be provided simultaneously until the infants were considered to have a stable breathing pattern (with regards to respiration, HR, and oxygen saturation and the oxygen concentration reaching a certain target value). In their study, the average cord clamping time was 5:49 ± 2:37 min in the intervention group, which was much longer than the median DCC time in our study. Nevertheless, their intervention group using a physiological-based cord clamping procedure was also unable to show any significant benefits in the short-term outcomes of the preterm infants.

In terms of the effect of placental blood transfusion, there was no significant difference in the peak Hct in the first 12–24 h, the peak bilirubin value, and the frequency of phototherapy between the two groups. Theoretically, the respiratory support was in positive correlation with placental blood transfusion during DCC. As a result, facilitating respiration would possibly increase the placental transfusion. However, in our study, there was no difference in the respiration-related indicators and the average duration of DCC in both groups. Therefore, this might have some relationship with being no difference with respect to placental transfusion. However, it was worth noting that, in our research, the ABG pH at 1 h after birth in the DCC+nCPAP group was 7.28 ± 0.08 vs. 7.25 ± 0.07 in the control group with the *p*-value standing of 0.052 (mean difference = 0.01, 95% CI: −0.01–0.05). This approached statistical significance (Table 3). An expanded sample size including more infants with gestational ages of <28 weeks will be needed in order to obtain more reliable evidence as to whether DCC+nCPAP could improve the placental blood transfusion during the early stage after birth.

In the short-term neonatal outcomes, the following results were not significantly different between the two groups: late-onset sepsis, IVH (≥grade 3), PVL, BPD (all grade), ROP (≥phase 2), NEC (≥phase 2), and death. The study by Katheria et al. (22) also found that with respect to preterm infants with a gestational ages of 23^0^-31^6^, the results were not significantly different between their ventilation during delayed cord clamping (V-DCC) group and the DCC-only group in the short-term neonatal outcomes such as admission temperature, peak bilirubin, morbidity of severe IVH/PVL/NEC, sepsis, and death (22). However, Polglase et al. (26) demonstrated that if ventilation was provided before cord clamping, the cerebral oxygenation of preterm lambs would be improved so as to prevent the incidence of severe IVH caused by sudden changes in cerebral perfusion pressure. While our study did not find a difference in the incidence of severe IVH between the two groups, their study was carried out under the condition of endotracheal intubation, which was different from our non-invasive ventilation. Endotracheal intubation had higher ventilation efficiency than non-invasive ventilation and avoided the influence of apnea and upper respiratory tract obstruction on respiration; this might be one of the reasons for the different results.

As for the safety of DCC+nCPAP, the meta-analysis showed that low oxygen saturation (<80%) and bradycardia (<100 bpm) within 5 min after birth were significantly correlated with death (27). Therefore, we included the incidence rate of SpO_2_ <80% and HR <100 bpm at 5 min after birth into the safety evaluation as indicators. One study showed that for infants with the admission temperature below 36°C, every decrease of 1°C in the body temperature would indicate an increase of 28% in mortality (28). Therefore, we used a rectal temperature of <36°C at admission as a safety evaluation indicator. In addition, we included maternal postpartum blood loss (29) and the incidence rate of postpartum infection as maternal safety evaluation indicators.

There were no significant statistical differences in the above indicators between the two groups. Furthermore, in the DCC+nCPAP group, the incidence rate of SpO_2_ <80% within 5 min after birth was 15.00%; the postpartum infection and hemorrhage rate were both 2.5%, which were lower than the incidence rates (46, 4, and 4%, respectively) reported in the literatures (21, 27, 30). There was only one infant with HR <100 bpm at 5 min after birth. Therefore, in our research, the safety of the HR was guaranteed. The incidence rate of infants' rectal temperature of <36.0°C at admission in our study was 0. Therefore, we conclude that DCC+nCPAP was a relatively safe program for preterm infants and mothers.

In addition, it should be noted that the mean admission temperature of infants in the two groups was ≥36.5°C. Our study also found that when DCC+ nCPAP was performed in a short period of time, even when the cart with heating gel and overhead heater specially designed for cord intact resuscitation mentioned by some researchers in their reports was not available, the infant's body temperature could also be well maintained without increasing the mother's risk of infection by raising the delivery room temperature, and drying and covering the infants with pre-heated towel.

The limitations of our research were as follows. (1) First, as mentioned above, most of the infants in our study had a gestational age >28 weeks, and the requirement for early respiratory support and the morbidity and mortality rates were relatively low. This might be a reason for the lack of any significant differences in clinical results. (2) Due to equipment limitations, we did not accurately monitor the time point of first and stable breathing after the birth of the infants. In addition, we were unable to obtain direct dynamic data including cerebral oxygen, cardiac function, and systemic blood flow during the early period after birth. This hindered us from evaluating the interaction between the establishment of stable spontaneous breathing and placental transfusion and the eventual outcome. It is our intention to include those parameters in a future study. Third, the contractility of the uterine smooth muscle was different between vaginal delivery and cesarean section; it would be better if the stratified randomization had been used during allocation. Fourth, this RCT did not demonstrate a significant difference in clinical outcome between the two groups; therefore, the sample size was a limiting factor.

## Conclusions

Our study showed that delayed cord clamping with nCPAP was feasible and safe in preterm infants with gestational age <32 weeks. Although there was a trend toward a higher arterial blood gas pH at 1 h after birth in the DCC+nCPAP group, DCC+ nCPAP neither resulted in a corresponding measurable clinical improvement nor did it reduce subsequent neonatal morbidity. A larger multi-center study including more infants with gestational age <28 weeks is needed to evaluate the full effects of DCC in combination with nCPAP in preterm infants.

## Data Availability Statement

The original contributions presented in the study are included in the article/Supplementary Material, further inquiries can be directed to the corresponding author.

## Ethics Statement

The studies involving human participants were reviewed and approved by Ethics Committee of the Chongqing Health Center for Women and Children. Written informed consent to participate in this study was provided by the participants' legal guardian/next of kin.

## Author Contributions

XZ initiated and managed the study, recruited the participants, performed the data analysis, data interpretation, writing, and revision. RD drafted the manuscript, participated in designing the study and was responsible for the inclusion and exclusion of studies, assessment of methodological quality, data analysis, writing, and revision. YW participated in designing the study, contributed to obtaining the grant, interpreted the data, and edited the manuscript. GX was responsible for data extraction, statistical analysis, and the manuscript editing. HG and WC were responsible for recruiting the participants, quality control, and the manuscript editing. LZ, BS, and QW assisted in the data analysis and the editing the manuscript. All the authors contributed to revision of the manuscript and approved the final version.

## Funding

This study was supported by the Natural Science Foundation of Chongqing, China, with Grant Numbers (cstc2020jcyj-msxmX0997 and cstc2020jcyj-msxmX0480).

## Conflict of Interest

The authors declare that the research was conducted in the absence of any commercial or financial relationships that could be construed as a potential conflict of interest.

## Publisher's Note

All claims expressed in this article are solely those of the authors and do not necessarily represent those of their affiliated organizations, or those of the publisher, the editors and the reviewers. Any product that may be evaluated in this article, or claim that may be made by its manufacturer, is not guaranteed or endorsed by the publisher.

## Abbreviations

ICC: immediate cord clamping
DCC: delayed cord clamping
IVH: intraventricular hemorrhage
NEC: necrotizing enterocolitis
ILCOR: International Liaison Committee on Resuscitation
PEEP: positive end-respiratory pressure
nCPAP: nasal continuous positive airway pressure
FRC: functional residual capacity
FiO_2_: fractional of inspiration O_2_
MV: mechanical ventilation
RCT: randomized controlled trial
PPV: positive pressure ventilation
PIP: peak inspiratory pressure
LISA: less-invasive surfactant administration
INSURE: intubate–surfactant–extubate
NICU: neonatal intensive care unit
SpO_2_: oxygen saturation
PaO_2_: arterial oxygen pressure
PS: pulmonary surfactant
RDS: respiratory distress syndrome
PCO_2_: partial pressure of carbon dioxide
HR: heart rate
BPD: bronchopulmonary dysplasia
PVL: periventricular leukomalacia
ROP: retinopathy of prematurity
PMA: postmenstrual age
BPM: beats per minute
pH: hydrogen ion concentration
Lac: lactate
Hct: hematocrit.
