# Supplementary figures and images for: With or Without Nasal Continuous Positive Airway Pressure During Delayed Cord Clamping in Premature Infants <32 Weeks: A Randomized Controlled Trial Using an Intention-To-Treat Analysis
Source: Front Pediatr. 2022 Mar 31;10:843372. doi: 10.3389/fped.2022.843372 (PMC9008252; doi:10.3389/fped.2022.843372)

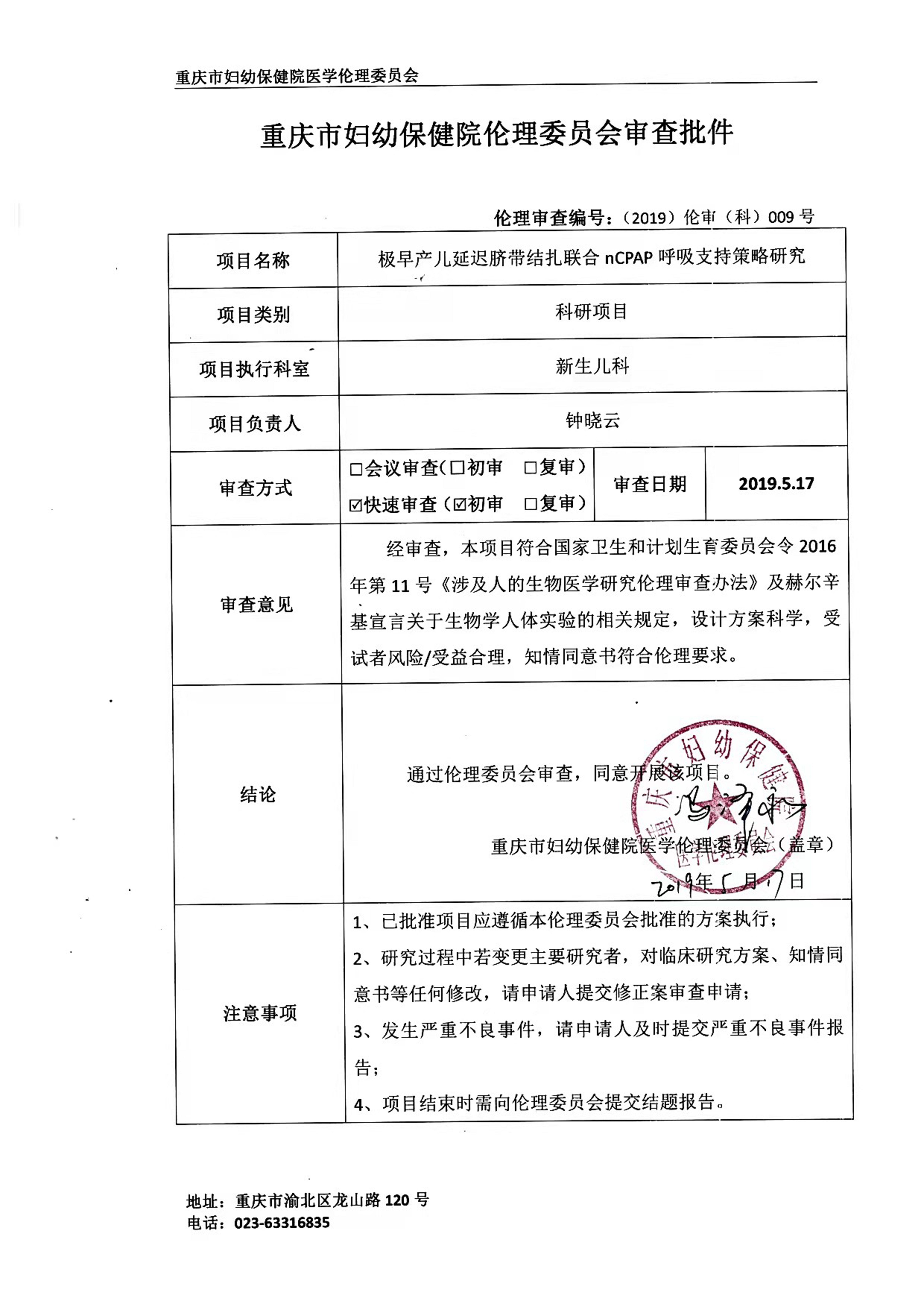

Supplement: Supplementary file 1 [file Image_1.JPEG]
